# Accurate Nonstandard Path Integral Models for Arbitrary Dielectric Boundaries in 2-D NS-FDTD Domains

**DOI:** 10.3390/s24072373

**Published:** 2024-04-08

**Authors:** Tadao Ohtani, Yasushi Kanai, Nikolaos V. Kantartzis

**Affiliations:** 1 Independent Researcher, Asahikawa 070-0841, Japan; bytcg100@ybb.ne.jp; 2 Department of Engineering, Faculty of Engineering, Niigata Institute of Technology, Kashiwazaki 945-1195, Japan; kanai@iee.niit.ac.jp; 3 School of Electrical and Computer Engineering, Faculty of Engineering, Aristotle University of Thessaloniki, 541 24 Thessaloniki, Greece

**Keywords:** electromagnetic analysis, finite-difference time-domain methods, integral equations, numerical analysis, radar cross section

## Abstract

An efficient path integral (PI) model for the accurate analysis of curved dielectric structures on coarse grids via the two-dimensional nonstandard finite-difference time-domain (NS-FDTD) technique is introduced in this paper. In contrast to previous PI implementations of the perfectly electric conductor case, which accommodates orthogonal cells in the vicinity of curved surfaces, the novel PI model employs the occupation ratio of dielectrics in the necessary cells, providing thus a straightforward and instructive means to treat an assortment of practical applications. For its verification, the reflection from a flat plate and the scattering from a cylinder using the PI model are investigated. Results indicate that the featured methodology can enable the reliable and precise modeling of arbitrarily shaped dielectrics in the NS-FDTD algorithm on coarse grids.

## 1. Introduction

The nonstandard finite-difference time-domain (NS-FDTD) method is a high-accuracy analysis tool of the finite-difference (FD) type, proposed by Cole [1,2,3], to improve the performance of the FDTD algorithm [4,5]. The scheme, based on an FD Laplacian featured via the nonstandard (NS) concept, introduced by Mickens [6], can reduce the overall propagation error of a typical FDTD implementation by a factor of $10^{-4}$ on a coarse grid at a desired frequency [1,2]. Through these excellent characteristics, the NS-FDTD method is particularly suitable for the accurate computation of the radar cross section (RCS) of electrically large objects. Amid the most prominent applications of the NS-FDTD method, one may discern the full-scale analysis of aircrafts—whose electrical size is very large, namely, more than 500 λ (λ is the wavelength)—with partially non-metal parts, including the radome, the canopy, the windows, and several radar-absorbing materials.

Nevertheless, the NS-FDTD method uses discrete space points to simulate electromagnetic wave propagation on orthogonal grids as in the FDTD technique [1,2,3,4,5]. Therefore, the discrete space treatment is unsuitable for realistic problems with arbitrarily shaped objects and fine details, not aligned to the grid axes [4,5,7,8,9,10,11], owing to the use of the insufficient staircase approximation on orthogonal grids in an effort to model the realistic object under study. Such structures can be, frequently, encountered in various applications, ranging from electromagnetic compatibility configurations [12,13,14] and microwave devices [15,16,17] to antennas [18,19,20], optical arrangements [21,22,23,24,25], and designs of low observability, including RCS scenarios. To circumvent such a drawback, a path integral (PI) model, based on the path integral form of Ampere’s and Faraday’s laws, has been previously presented [26,27,28]. The specific path integral scheme can effectively handle real-world objects on orthogonal meshes, as the integral path may be intuitively placed on the object’s surface. Thus, and owing to the PI model, the applicability of the NS-FDTD technique can be drastically expanded. However, the prior technique has been formulated only for the case of perfectly electric conductors (PECs); a fact that prohibits the analysis of various dielectric components found in many contemporary avionic and microwave telecommunication systems.

In this paper, a robust PI model for the rigorous treatment of dielectric objects, whose shape does not fit to any orthogonal grid, via the 2-D NS-FDTD method is developed. The proposed PI concept launches a simple scheme that considers the occupation ratio of all dielectrics at a specific cell. In this way, the manipulation of realistic dielectric structures becomes feasible on a coarse lattice. Firstly, to certify the key competence of the proposed PI model, we perform a reflection analysis of a flat plate and compare it with the exact solution. Next, an RCS problem is comprehensively explored via a real-world application. It is emphasized that for the NS-FDTD method, a practical grid width, Δ, is Δ≤λ/8. On the other hand, if dielectrics are involved in the computational domain, the modified grid width should be $\Delta^{\prime }\leq \lambda /\left(8\sqrt{\varepsilon_{r}}\right)$ ($\varepsilon_{r}$ is the relative dielectric permittivity). Furthermore, the use of a much finer lattice, i.e., $\Delta^{\prime }\ll \Delta$, in order to satisfactorily treat the shape of a complicated structure, poses prohibitive issues in the implementation of the NS-FDTD method due to the unduly increase of the necessary system memory requirements and the extremely prolonged simulation times. These considerable drawbacks can be drastically circumvented by means of the proposed PI method, without the need for any additional conventions or non-physical assumptions, as occurs in existing schemes. Finally, all numerical outcomes reveal that the featured technique exhibits superior accuracy and convergence, as opposed to other computational approaches (with much finer spatial resolutions), for the trustworthy study of dielectric devices with intricate shape and elaborate geometrical features.

## 2. The PI Model at a Dielectric Boundary

### 2.1. Basics and Formulation

**Figure 1:**
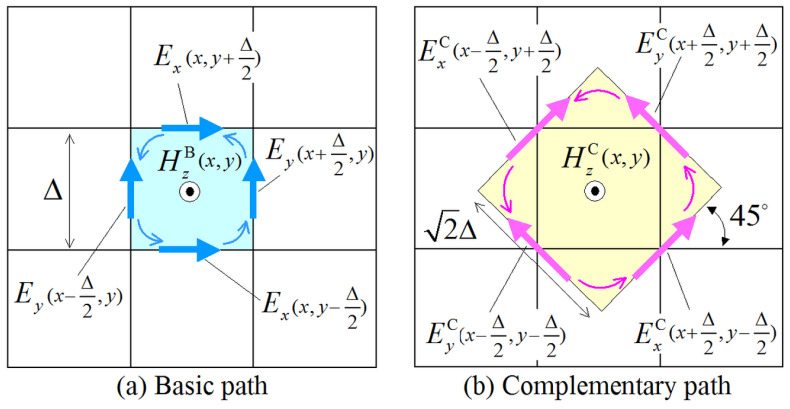
The two paths—(**a**) basic and (**b**) complementary—involved in the proposed integral NS-FDTD form for the calculation $H_{z}$ magnetic-field component on a square lattice. The two paths are rotated $45^{\circ }$, one with regard to the other, in order to mutually cancel their discretization errors.

**Figure 2:**
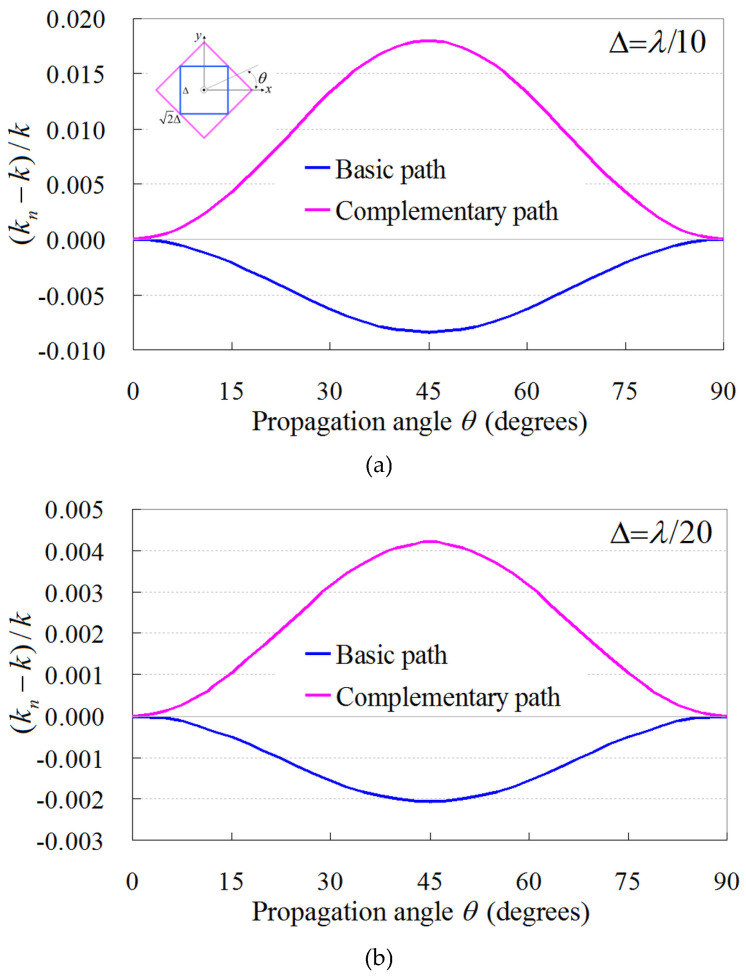
Propagation accuracy of the basic and complementary PI model using the nonstandard correction function $s_{k}\left(\Delta \right)=2\sin\left(k\Delta /2\right)/k$ for (**a**) Δ=λ/10 and (**b**) Δ=λ/20.

First, let us review the concept of the PI model for the NS-FDTD method [26,27,28]. The model uses both basic and complementary paths, as depicted in Figure 1, for the evaluation of the $H_{z}$ magnetic-field component. Herein, we intend to establish a complementary relation between these two paths. The idea is based on the wave propagation characteristics of the Yee algorithm [1,2,5], where maximum and minimum errors occur at an angle of $45^{\circ }$ on a square lattice. This indicates that we can mutually cancel these errors by means of two meshes for one node calculation. In an effort to attain the optimal cancellation of the inevitable discretization errors, the complementary (C) path is rotated $45^{\circ }$ with respect to the basic (B) one. Specifically, the two integral paths are expressed as

(1) $$\begin{array}{cc} \mu \int \frac{\partial H}{\partial t} & \cdot dS=-\int E\cdot dl\Rightarrow \mu \frac{\partial H_{z}^{B}\left(x,y\right)}{\partial t}\Delta^{2}\\ \; & =-\left[E_{y}\left(x+\Delta /2,y\right)-E_{x}\left(x,y+\Delta /2\right)-E_{y}\left(x-\Delta /2,y\right)+E_{x}\left(x,y-\Delta /2\right)\right]\Delta , \end{array}$$

for the basic path, with $S=\Delta^{2}$ (Figure 1a), and

(2) $$\begin{array}{cc} \mu \frac{\partial H_{z}^{C}\left(x,y\right)}{\partial t}2\Delta^{2}= & -\left[E_{y}^{C}\left(x+\Delta /2,y+\Delta /2\right)-E_{x}^{C}\left(x-\Delta /2,y+\Delta /2\right)\right.\\ \; & \left.-E_{y}^{C}\left(x-\Delta /2,y-\Delta /2\right)+E_{x}^{C}\left(x+\Delta /2,y-\Delta /2\right)\right]\sqrt{2}\Delta , \end{array}$$

for the complementary path (Figure 1b). Note that in (1) and (2), we have replaced Δ with the nonstandard correction function $s_{k}\left(\Delta \right)=2\sin\left(k\Delta /2\right)/k$, where *k* is the physical wavenumber [1,2]. Based on this substitution, the propagation accuracy, $(k_{n}-k)/k$, of the basic and complementary path model is shown in Figure 2 for two lattice resolutions and $k_{n}$, representing the numerical wavenumber of the proposed PI model. The results are obtained via the method presented in [3,27], and as can be promptly observed, both models exhibit opposite propagation errors for the desired mutual cancellation. In this manner, the new PI model is derived by means of

(3) $$\begin{array}{c} \frac{\partial H_{z}\left(x,y\right)}{\partial t}=\beta_{0}\frac{\partial H_{z}^{B}\left(x,y\right)}{\partial t}+\left(1-\beta_{0}\right)\frac{\partial H_{z}^{C}\left(x,y\right)}{\partial t}, \end{array}$$

where, for homogeneous media, $\beta_{0}$ is given by [26]

(4) $$\begin{array}{c} \beta_{0}\approx \frac{2}{3}-\frac{{\left(k\Delta \right)}^{2}}{90}. \end{array}$$

On the dielectric surface, we select the first term of (4), i.e., $\beta_{0}=2/3$, which is independent of the *k*, as the latter varies across the dielectric boundary. Additionally, in (3), we consider $\partial H_{z}/\partial t=\left(H_{z}^{n+1}-H_{z}^{n}\right)/s_{\omega }\left(\Delta t\right)$, with $s_{\omega }\left(\Delta t\right)=2\sin\left(\omega \Delta /2\right)/\omega$, from the nonstandard formulation of [1,2], ω is the angular frequency, Δt is the time step, and n=t/Δt. On the other hand, the $E_{y}^{C}$ term in (2) is obtained from the already known $E_{x,y}$ values on the basic path as

(5) $$\begin{array}{cc} E_{y}^{C}\left(x+\Delta /2,y+\Delta /2\right) & =\frac{1}{2}\left\{\left[E_{x}\left(x,y+\Delta /2\right)+E_{x}\left(x+\Delta ,y+\Delta /2\right)\right]e_{x}\right.\\ \; & \left.+\left[E_{y}\left(x+\Delta /2,y\right)+E_{y}\left(x+\Delta /2,y+\Delta \right)\right]e_{y}\right\}\cdot \frac{-e_{x}+e_{y}}{\sqrt{2}}, \end{array}$$

where $e_{x,y}$ are the corresponding unit vectors. Bear in mind that an analogous expression for the $E_{x}^{C}$ term can be similarly obtained.

### 2.2. Treatment of the Magnetic-Field Components

**Figure 3:**
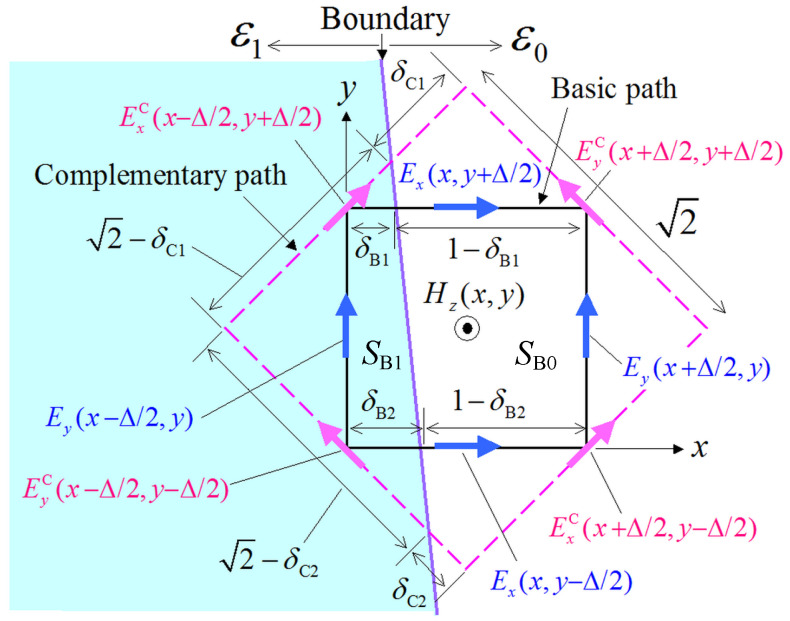
The proposed PI model for the calculation of the $H_{z}$ component at a Δ×Δ lattice in the case of a dielectric boundary between an $\varepsilon_{0}$ and an $\varepsilon_{1}$ medium. The path length is measured via the $s_{k}\left(\Delta \right)$ nonstandard correction function [1,2]. Moreover, $S_{B0,B1}$ are the areas occupied by the $\varepsilon_{0,1}$ dielectric medium inside the basic path, $\delta_{B1,B2}$ are path lengths along the basic path in the $\varepsilon_{1}$ medium, and $\delta_{C1,C2}$ are path lengths along the complementary path in the $\varepsilon_{0}$ medium.

The new PI model of (1) and (2) is, now, extended to the treatment of the definitely more demanding dielectric boundaries. Therefore, based on the geometric depiction of the proposed PI for such a scenario, the μ∫(∂H/∂t)·dS=−∫E·dl along the basic path in Figure 3 is given by

(6) $$\begin{array}{cc} \mu \frac{\partial H_{z}^{B}}{\partial t}\left(S_{B0}+S_{B1}\right)= & -\left[E_{y}\left(x+\Delta /2,y\right)l_{0}-E_{y}\left(x-\Delta /2,y\right)l_{1}\right.\\ \; & -E_{x}{\left(x,y+\Delta /2\right)}_{\varepsilon_{0}}\left(1-\delta_{B1}\right)l_{0}-E_{x}{\left(x,y+\Delta /2\right)}_{\varepsilon_{1}}\delta_{B1}l_{1}\\ \; & \left.+E_{x}{\left(x,y-\Delta /2\right)}_{\varepsilon_{0}}\left(1-\delta_{B2}\right)l_{0}+E_{x}{\left(x,y-\Delta /2\right)}_{\varepsilon_{1}}\delta_{B2}l_{1}\right], \end{array}$$

where $l_{0,1}\equiv s_{k_{0,1}}\left(\Delta \right)=2\sin\left(k_{0,1}\Delta /2\right)/k_{0,1}$ with $k_{0,1}$ as the wavenumber of the $\varepsilon_{0,1}$ dielectric medium, $S_{B0,B1}$ are the areas occupied by the $\varepsilon_{0,1}$ dielectric medium inside the basic path, and $\delta_{B1,B2}$ are path lengths along the basic path in the $\varepsilon_{1}$ medium. Also, the $E_{x,y}{\left(x,y\right)}_{\varepsilon_{0,1}}$ notation refers to the $E_{x,y}$ component in the $\varepsilon_{0,1}$ dielectric medium. Note that if $E_{x,y}$ is not available at a specific node on the grid, we use the $E_{x,y}$ extrapolation values at the nearest-neighbor node; for example, $E_{x}{\left(x,y+\Delta /2\right)}_{\varepsilon_{1}}∼E_{x}\left(x-\Delta ,y+\Delta /2\right)$. Similarly, the $\partial H_{z}^{C}/\partial t$ term using the complementary path in Figure 3 is given by

(7) $$\begin{array}{cc} \mu \frac{\partial H_{z}^{C}}{\partial t} & \left(S_{C0}+S_{C1}\right)=-\left[E_{y}^{C}{\left(x+\Delta /2,y+\Delta /2\right)}_{\varepsilon_{1}}\sqrt{2}l_{0}-E_{y}^{C}{\left(x-\Delta /2,y-\Delta /2\right)}_{\varepsilon_{0}}\delta_{C2}l_{0}\right.\\ \; & -E_{y}^{C}{\left(x-\Delta /2,y-\Delta /2\right)}_{\varepsilon_{1}}\left(\sqrt{2}-\delta_{C2}\right)l_{1}-E_{x}^{C}{\left(x-\Delta /2,y+\Delta /2\right)}_{\varepsilon_{1}}\left(\sqrt{2}-\delta_{C1}\right)l_{1}\\ \; & \left.-E_{x}^{C}{\left(x-\Delta /2,y+\Delta /2\right)}_{\varepsilon_{0}}\delta_{C1}l_{0}+E_{x}^{C}{\left(x+\Delta /2,y-\Delta /2\right)}_{\varepsilon_{1}}l_{0}\right]. \end{array}$$

It should be stressed that for the practical application of (7), we assume that the $E_{x,y}^{C}{\left(x,y\right)}_{\varepsilon_{0,1}}$ terms exist on the integral path that straddles the dielectric boundary. In this manner, we can project the already known $E_{x,y}\left(x,y\right)$ values to the corresponding $e_{x,y}$ directional unit vector on the integral path. For illustration, the $E_{x}^{C}{\left(x-\Delta /2,y+\Delta /2\right)}_{\varepsilon_{0}}$ term in (7) is obtained from the $E_{x}\left(x,y+\Delta /2\right)e_{x}\cdot \left(e_{x}+e_{y}\right)/\sqrt{2}$ projection. Then, and similarly to (5), the $E_{x}^{C}{\left(x-\Delta /2,y+\Delta /2\right)}_{\varepsilon_{1}}$ term in (7) is acquired from the inner product of $\left\{E_{x}\left(x-\Delta ,y+\Delta /2\right)e_{x}+\left[E_{y}\left(x-\Delta /2,y\right)+E_{y}\left(x-\Delta /2,y+\Delta \right)\right]e_{y}/2\right\}\cdot \left(e_{x}+e_{y}\right)/\sqrt{2}$, with an analogous treatment holding for the other $E^{C}$ terms, as well. Having determined the $\partial H_{z}^{B}/\partial t$ and $\partial H_{z}^{C}/\partial t$ quantities from (6) and (7), respectively, the required $\partial H_{z}/\partial t$ derivative is evaluated by means of (3).

### 2.3. Treatment of the Electric-Field Components

**Figure 4:**
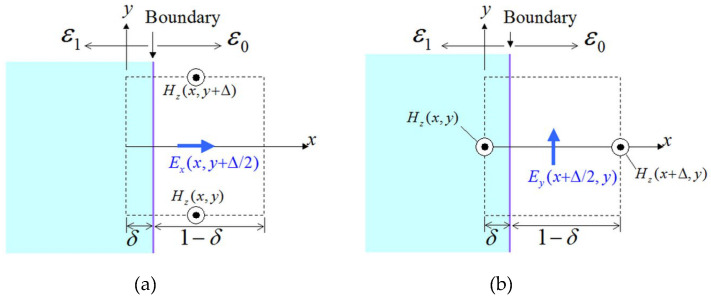
Treatment of a (**a**) normal $E_{x}$ component and (**b**) parallel $E_{y}$ component with respect to the dielectric boundary. Here, δ is the occupation ratio equal to the area of the $\varepsilon_{1}$ medium over the entire area of a cell.

The most frequently encountered cases for the computation of electric-field quantities at a dielectric boundary are those of a normal $E_{x}$ and a parallel $E_{y}$ component with regard to the boundary, as described in Figure 4. In particular, concentrating on Figure 4a and in terms of ε∫(∂E/∂t)·dS=∫H·dl, we derive

(8) $$\begin{array}{c} E_{x}^{n+1/2}\left(x,y+\Delta /2\right)=E_{x}^{n-1/2}\left(x,y+\Delta /2\right)+{\mathrm{ch}}_{n}\left[H_{z}^{n}\left(x,y+\Delta \right)-H_{z}^{n}\left(x,y\right)\right], \end{array}$$

for

(9) $$\begin{array}{c} {\mathrm{ch}}_{n}=\delta {\mathrm{ch}}_{1}+\left(1-\delta \right){\mathrm{ch}}_{0},\;\;{\mathrm{ch}}_{1}=\frac{s_{\omega }\left(\Delta t\right)}{\varepsilon_{1}s_{k_{1}}\left(\Delta \right)},\;\;{\mathrm{ch}}_{0}=\frac{s_{\omega }\left(\Delta t\right)}{\varepsilon_{0}s_{k_{0}}\left(\Delta \right)}. \end{array}$$

Moreover, for the scenario of Figure 4b, one acquires

(10) $$\begin{array}{c} E_{y}^{n+1/2}\left(x\;+\;\Delta /2,y\right)\;=\;E_{y}^{n-1/2}\left(x\;+\;\Delta /2,y\right)\;-\;{\mathrm{ch}}_{p}\left[H_{z}^{n}\left(x\;+\;\Delta ,y\right)s_{k_{0}}\left(\Delta \right)\;-\;H_{z}^{n}\left(x,y\right)s_{k_{1}}\left(\Delta \right)\right], \end{array}$$

for

(11) $$\begin{array}{c} {\mathrm{ch}}_{p}=\frac{s_{\omega }\left(\Delta t\right)}{\delta \varepsilon_{1}s_{k_{1}}^{2}\left(\Delta \right)+\left(1-\delta \right)\varepsilon_{0}s_{k_{0}}^{2}\left(\Delta \right)}. \end{array}$$

**Figure 5:**
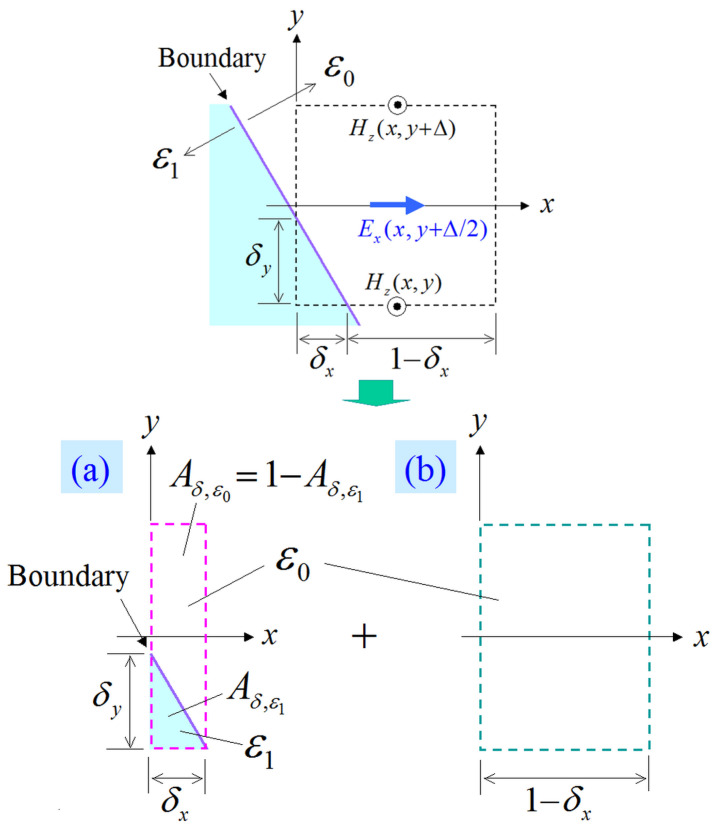
Division model of an $E_{x}$ cell to treat an oblique dielectric boundary. The boundary case for the $E_{x}$ cell is divided into (**a**) the parallel case and (**b**) the free-space case.

On the other hand, when the case of an obliquely aligned, with respect to the lattice, dielectric boundary is taken into account, we introduce the cell division scheme of Figure 5, where $A_{\delta ,\varepsilon }$ is the occupied area ratio. Explicitly, Figure 5a is considered as the parallel case of Figure 4b, while the entire model in Figure 5a,b is treated as the normal case of Figure 4a. Hence, the corresponding $\partial E_{x}/\partial t$ can be expressed as

(12) $$\begin{array}{cc} E_{x}^{n+1/2}\left(x,y+\Delta /2\right)=E_{x}^{n-1/2}\left(x,y+\Delta /2\right) & -\delta_{x}{\mathrm{ch}}_{a}\left[H_{z}^{n}\left(x,y+\Delta \right)s_{k_{0}}\left(\Delta \right)-H_{z}^{n}\left(x,y\right)s_{k_{1}}\left(\Delta \right)\right]\\ \; & +\left(1-\delta_{x}\right){\mathrm{ch}}_{0}\left[H_{z}^{n}\left(x,y+\Delta \right)-H_{z}^{n}\left(x,y\right)\right], \end{array}$$

for

(13) $$\begin{array}{c} {\mathrm{ch}}_{a}=\frac{s_{\omega }\left(\Delta t\right)}{\varepsilon_{1}s_{k_{1}}\left(\Delta \right)A_{\delta ,\varepsilon_{1}}+\varepsilon_{0}s_{k_{0}}\left(\Delta \right)A_{\delta ,\varepsilon_{0}}}, \end{array}$$

with a similar formula holding for the $\partial E_{y}/\partial t$ temporal derivative. In addition, the $E_{x,y}^{C}$ components on the complementary path can be extracted through the inner products of these $E_{x,y}$ components with the respective unit vector $u_{x,y}=\left(e_{y}\pm e_{x}\right)/\sqrt{2}$. Notice that (8)–(13) are the best models, derived theoretically and numerically from the previous analysis. Finally, as a supplement, a special case of the right-angle edge can be treated by (i) selecting the Δ width, so that the edge fits on the grid, (ii) moving the edge to the grid line, so that it fits on, and (iii) applying the modified PI models of Figure 4 and Figure 5.

## 3. Numerical Results and Discussion

### 3.1. Reflectivity Analysis of a Flat Dielectric Plate

**Figure 6:**
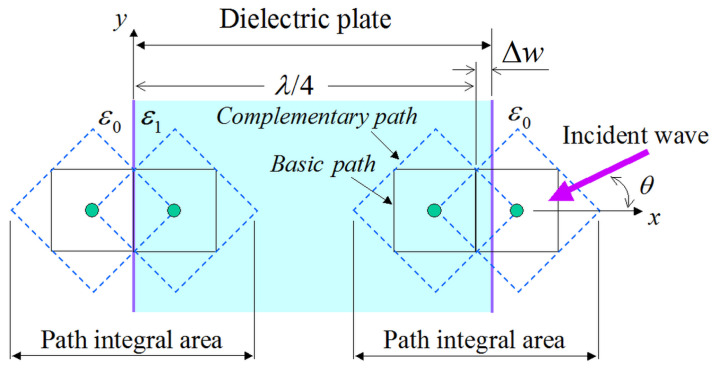
Reflectivity evaluation of a flat dielectric plate by means of the proposed PI model, where, except for the PI area, the rest of the domain is a differential type NS-FDTD region. Note that Δw is the distance between the grid line of the basic path and the plate edge, with Δw=0 at the left side. The dielectric plate is infinite along the ±y directions with a thickness of λ/4+Δw.

**Figure 7:**
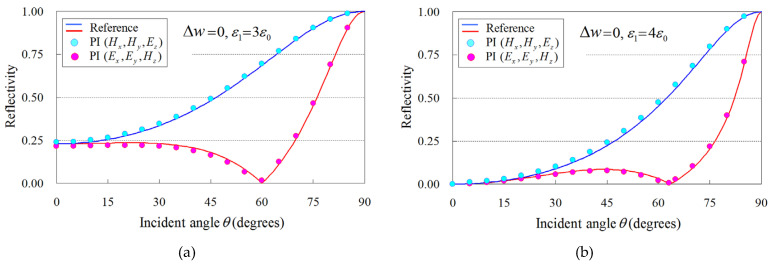

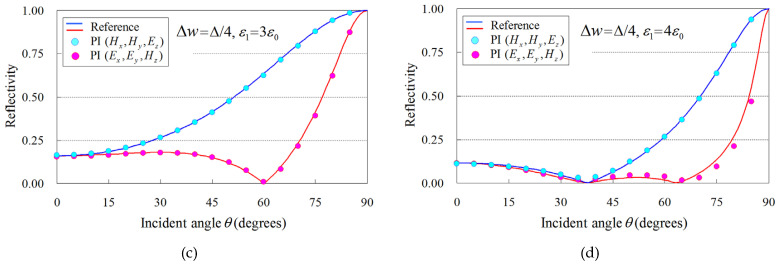
Reflectivity ($\mathrm{TM}=(H_{x},H_{y},E_{z})$ and $\mathrm{TE}=(E_{x},E_{y},H_{z})$) analysis versus the incidence angle, θ, for the flat dielectric plate of Figure 6, performed in terms of the proposed PI model and compared to the analytical (reference) solution of [31]. The analysis involves two dielectric media (acrylic resin and glass epoxy) in conjunction with two Δw arrangements (Δw=0: the right-hand side of the plate surface is aligned to the grid and Δw=Δ/4: the plate surface is not aligned to the grid). (**a**) Acrylic resin with $\varepsilon_{1}=3\varepsilon_{0}$ and Δw=0, (**b**) glass epoxy with $\varepsilon_{1}=4\varepsilon_{0}$ and Δw=0, (**c**) acrylic resin with $\varepsilon_{1}=3\varepsilon_{0}$ and Δw=Δ/4, and (**d**) glass epoxy with $\varepsilon_{1}=4\varepsilon_{0}$ and Δw=Δ/4.

In order to substantiate the merits of the featured PI model, we focus on the reflectivity analysis of a flat dielectric plate as a basic example with an analytic solution. The computational domain is shown in Figure 6, whereas the PI models of (5)–(13) are employed only on the dielectric surface. Moreover, the plate is infinite along the ±y directions, and an incident plane wave impinges from the right side in Figure 6. Therefore, the sine–cosine method [29] is used for our oblique incidence calculations. For our ($\mathrm{TM}=(H_{x},H_{y},E_{z})$ and $\mathrm{TE}=(E_{x},E_{y},H_{z})$) analysis, we examine two dielectric media, namely, an acrylic resin with $\varepsilon_{1}=3\varepsilon_{0}$ and a glass epoxy with $\varepsilon_{1}=4\varepsilon_{0}$. The remaining implementation aspects are: *λ* = 1 m, Δ=λ/20, and Δt=T/30, with *T* as the corresponding wave period, while open-space truncation is conducted in terms of a 15Δ-thick perfectly matched layer (PML) [30]. To facilitate our comparisons, we use, as our reference, the analytical solution of the specific problem described in [31]. In this context, Figure 7 illustrates the reflectivity of the flat dielectric plate for the aforementioned materials and two Δw configurations, i.e., Δw=0 (the right-hand side of the plate surface is aligned to the grid) and Δw=Δ/4 (the plate surface is not aligned to the grid). As observed, all the outcomes derived via the enhanced PI models are in promising agreement with the reference solution, thus proving the competence of the new scheme to effectively treat arbitrary dielectric boundaries. In this case, the flat plate model can be treated accurately for a geometrical integral path length and occupied ratio in the cell since the dielectric boundary is straight and parallel to the grid line (*y*-axis). Hence, we can detect that the PI model closely follows the analytic solution if we utilize an exact path length and occupied ratio in the PI cell. This fact proves the validity of the proposed PI model in the analysis of arbitrary dielectric boundaries.

### 3.2. RCS Analysis of a Dielectric Cylinder

**Figure 8:**
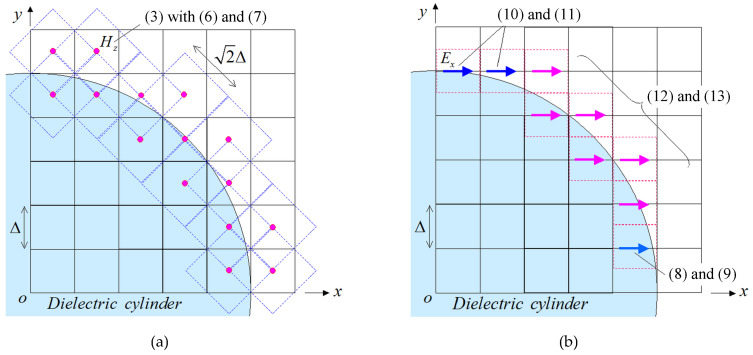
The PI model of an infinite dielectric cylinder (first quadrant) for the TE mode with the positions of the involved (**a**) $H_{z}$ components, computed through (3), (6), and (7), and (**b**) $E_{x}$ components, calculated via (8)–(13). The proposed scheme is used solely on the cylinder surface, while the remaining domain is a typical NS-FDTD region. Note that due to rotational symmetry, the $E_{y}$ case is similar to the $E_{x}$ one.

Probing further, we proceed to a more realistic application and compute the RCS of an infinite dielectric cylinder with a radius of 5Δ and a relative permittivity of $\varepsilon_{r}=3$. For the curved boundary, we utilize the oblique dielectric models of Figure 5, formulated in (6)–(13). The grid layout is given in Figure 8, where the novel PI model is applied only on the cylinder surface and the rest of the domain is a regular NS-FDTD region. In particular, Figure 8a depicts the PI model of the $H_{z}$ component, expressed via (3), (6), and (7), while Figure 8b presents the PI model for the $E_{x}$ component, described by (8)–(13). This configuration is attributed to the occupation ratio of the dielectrics and the direction of the electric field in the shaded (cyan) cells. Note that the treatment of the $E_{y}$ component is the same as the $E_{x}$ one, owing to the rotational symmetry of the problem. Furthermore, for the excitation of the structure through an incident plane wave (impinging with an angle of $\theta =45^{\circ }$) and the evaluation of the scattered waves from the dielectric cylinder, the total-field/scattered-field formulation [5] is employed. Finally, the scattered waves, so obtained, are converted to the appropriate RCS data by means of the near-to-far-field transformation technique [5,31].

**Figure 9:**
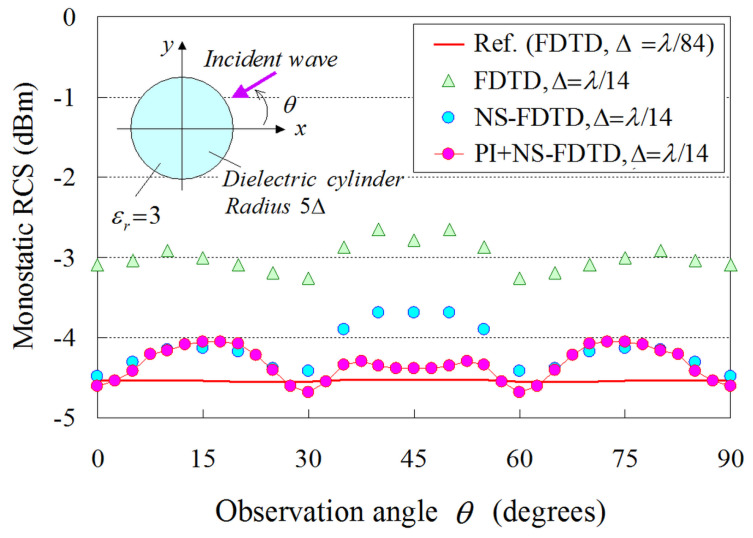
Monostatic RCS results derived from the proposed (PI+NS-FDTD) technique, the conventional FDTD method, and the NS-FDTD algorithm without the PI model. The reference solution is acquired from an FDTD realization with a very fine (Δ=λ/84, Δt=T/120) lattice resolution.

**Figure 10:**
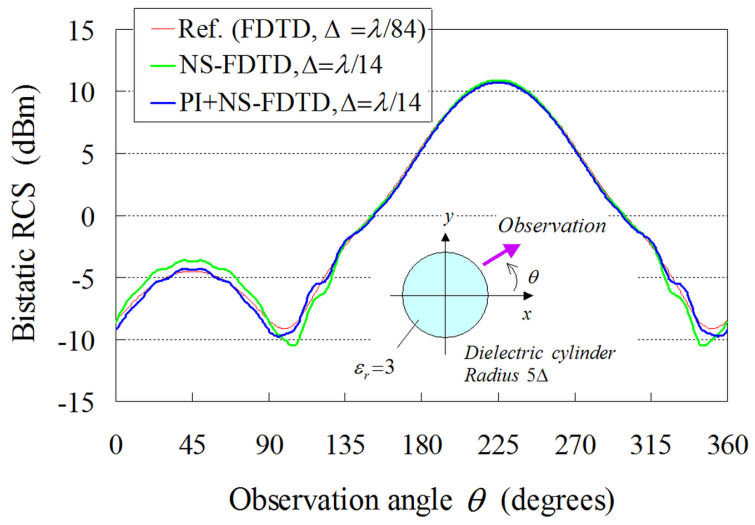
Bistatic RCS results derived from the proposed (PI+NS-FDTD) technique and the NS-FDTD algorithm without the PI model. The reference solution is acquired from an FDTD realization with a very fine (Δ=λ/84, Δt=T/120) lattice resolution. The incidence angle of the impinging plane wave is $45^{\circ }$, while the geometric PI cell data of the cylinder are depicted in Figure 8.

In this framework, Figure 9 illustrates the comparative results between our model (PI model combined with the NS-FDTD technique: PI+NS-FDTD) and other approaches (typical FDTD and NS-FDTD method without the PI model) for *λ* = 1 m, Δ=λ/14, and Δt=T/20. Also, the outcomes of a much finer FDTD implementation (Δ=λ/84, Δt=T/120) serve as our reference solution. It becomes apparent that the PI+NS-FDTD method agrees very well with the reference data within the range of 0.5 dBm, which is, actually, a negligible difference in most real-world RCS configurations. Herein, the reduction effect is ${\left(14/84\right)}^{2}$ for the overall computer memory and the same analysis space size since the reference FDTD solution uses a Δ=λ/84 and the NS-FDTD simulation with the PI model employs a Δ=λ/14. Additionally, our method achieves a reduction effect of ${\left(14/84\right)}^{2}\times \left(20/120\right)$ for the total CPU time, compared with the FDTD algorithm, because the reference FDTD solution uses a Δt=T/120, while the NS-FDTD simulation with the PI model employs a Δt=T/20. In Figure 9, a small fluctuation of 0.5 dBm is observed in the PI results for the observation angle θ. This small discrepancy can be attributed to the geometrical layout of the PI cell in Figure 8, as the PI models periodically vary regarding the cylinder surface position. To mitigate this RCS fluctuation, one may use finer cells, as deduced from the FDTD results with Δ=λ/14 and Δ=λ/84, since the specific PI model robustly replaces the complicated scattering phenomena on the cylinder surface (smaller than Δ×Δ) with one cell calculation on the coarse grid. However, it should be emphasized that the RCS fluctuation versus the observation angle is much smaller than that of the NS-FDTD simulation without the PI model. Lastly, regarding the bistatic RCS for $\theta =45^{\circ }$, our scheme offers, again, a very satisfactory performance, as shown in Figure 10, for the same set of implementation parameters as in the prior example. It should be stated that only a small RCS fluctuation is detected in the PI outcomes. Bear in mind that if a sufficiently fine (i.e., Δ=λ/20) cell is selected in Figure 8, such fluctuations are not at all observed. This implies that the fluctuations in Figure 10 are attributed to the use of the ${\left.\Delta =\lambda /(8\sqrt{\varepsilon_{r}})\right|}_{\varepsilon_{r}=3}∼\lambda /14$ maximum grid size [1,2] and the geometrical layout of the PI cell, as already mentioned in the monostatic case. Consequently, the novel formulation can reliably and accurately treat the curved shape of dielectric structures, even when their surface is not aligned to the axes of orthogonal NS-FDTD grids.

## 4. Conclusions

Conventional grid-based computational methods exhibit significant precision issues since they are not able to efficiently model curved media boundaries. To alleviate these shortcomings and accurately handle dielectric objects of an arbitrary shape, not aligned to the grid axes, a consistent and efficient PI form for the 2-D NS-FDTD technique has been presented in this paper. The key idea of the proposed PI scheme is to utilize the occupation ratio of dielectrics in the necessary cells, surrounded by basic and complementary paths, thus attaining a notable reduction of the overall system burden. Conducting the numerical RCS analysis of a dielectric plate and cylinder, it has been validated that the new concept improves the accuracy of the regular NS-FDTD algorithm in the case of dielectric boundaries, considerably curved interfaces, and fine geometric characteristics. Thus, the universality of the NS-FDTD technique can be seriously expanded, even on coarse grids, without having to resort to any additional complicated approaches or artificial assumptions. Specifically, for the demanding RCS study of electrically large configurations (partially occupied by various dielectrics), which opt for excessive memory requirements and elongated simulations, the new PI model is proven to offer remarkable computational savings. Nonetheless, it should be stated that generating the appropriate geometrical data for the PI model is a laborious task from a calculation point of view. Therefore, for a beneficial real-world use of the featured scheme, the development of a CAD tool is deemed necessary.

## Data Availability

Data are contained within the article.
